# Twitter Influence on UK Vaccination and Antiviral Uptake during the 2009 H1N1 Pandemic

**DOI:** 10.3389/fpubh.2016.00026

**Published:** 2016-02-22

**Authors:** Andrew McNeill, Peter R. Harris, Pam Briggs

**Affiliations:** ^1^Department of Psychology, Northumbria University, Newcastle Upon Tyne, UK; ^2^School of Psychology, University of Sussex, Brighton, UK

**Keywords:** social media, pandemic, influenza, public health, H1N1 virus

## Abstract

**Objective:**

Information exchange via Twitter and other forms of social media make public health communication more complex as citizens play an increasingly influential role in shaping acceptable or desired health behaviors. Taking the case of the 2009–2010 H1N1 pandemic, we explore in detail the dissemination of H1N1-related advice in the UK through Twitter to see how it was used to discourage or encourage vaccine and antiviral uptake.

**Methods:**

In three stages we conducted (1) an analysis of general content, retweeting patterns, and URL sharing, (2) a discourse analysis of the public evaluation of press releases and (3) a template analysis of conversations around vaccine and antiviral uptake, using Protection Motivation Theory (PMT) as a way of understanding how the public weighed the costs and benefits.

**Results:**

Network analysis of retweets showed that information from official sources predominated. Analysing the spread of significant messages through Twitter showed that most content was descriptive but there was some criticism of health authorities. A detailed analysis of responses to press releases revealed some scepticism over the economic beneficiaries of vaccination, that served to undermine public trust. Finally, the conversational analysis showed the influence of peers when weighing up the risks and benefits of medication.

**Conclusion:**

Most tweets linked to reliable sources, however Twitter was used to discuss both individual and health authority motivations to vaccinate. The PMT framework describes the ways individuals assessed the threat of the H1N1 pandemic, weighing this against the perceived cost of taking medication. These findings offer some valuable insights for social media communication practices in future pandemics.

## Introduction

Pandemics pose a challenge to public health officials, who need to coordinate a swift and effective communication strategy so that the general public can be informed about the risks of the pandemic and the appropriate behavioral response to those risks. A failure to communicate effectively at such times can be very serious (1). In this paper, we add to the body of knowledge about the role of social media in communicating information about a pandemic, focusing upon the UK response to the H1N1 virus.

H1N1 was an influenza virus that originated in Mexico. In April 2009, the World Health Organisation (WHO) announced that they had detected the rapid spread of this virus and public health bodies worldwide began to make preparations (2). During this period, social media sites were used to communicate information and thoughts about the pandemic and how to deal with it, which meant that, for the first time, the pandemic could be explored through the analysis of social media networks in general, and the analysis of Twitter in particular (3). Indeed, Chew and Eysenbach (3) described the H1N1 pandemic as occurring in the “Age of Twitter.”

The control of health messages during a pandemic has never lain entirely in the hands of health professionals. Word of mouth has always been important and the press and broadcast news media have been shown to have a strong role in influencing public opinion and behavior during earlier pandemics, such as SARS (4, 5). While the influence of mainstream media has continued to be a focus for research (6, 7), a number of recent studies have explored the democratization of influence that comes with Twitter and other forms of social media (8).

This “democratization” brings both challenges and opportunities for the health community. For example, consider the roll-out of public vaccination programs. On the one hand, there are new opportunities for health authorities to engage directly with the general public or to target vulnerable groups with carefully customized information about appropriate vaccination or antiviral use. On the other hand, there are new and plentiful opportunities for dissent. For example, antivaccination groups, who may previously have had a limited sphere of influence, gain a new voice in social media and acquire the opportunity to be heard alongside official health advice. Private concerns about vaccination can be spread to thousands of followers in an instant – increasing for many the perception of the risk of vaccinating.

The role of Twitter in the 2009–2010 H1N1 (“swine flu”) pandemic has been studied in some detail, in part because the pandemic emerged just at a time when Twitter (established in 2006) was becoming popular. The largest study is that of Chew and Eysenbach (3) who took large samples of data globally during the pandemic and explored the kinds of content being shared (such as resources, personal experiences, opinions and marketing). They found that the largest category of information was “resource” information (i.e., sharing of descriptive information, usually with links to other websites). Nevertheless, personal experience and opinion made up 32% of the tweets. They were able to show temporal trends in sentiment, misinformation, and expressions of personal opinion, showing how Twitter could be a useful source of data for understanding public reactions during pandemics. Furthermore, such sentiment-coded data can be used to predict the uptake of vaccines in specific areas depending on the level of sentiment expressed in tweets from that area (9). Information about the vaccine tended to be shared between users who shared similar sentiment (9) and having larger numbers of opinionated friends on Twitter tended to inhibit expressing sentiment about the vaccine (10). However, the presence of negative sentiment in tweets about the vaccine tended to breed future negative sentiment from other connected users, showing the contagious nature of negative sentiment (10). Other research (11) showed that Twitter users had a preference for sharing websites that contained reliable information (e.g., sites like BBC, WHO, or CDC) although in some circumstances unreliable information was prominent. Few other studies specifically study the content of Twitter messages during pandemics, although several studies have pointed out the value of Twitter for predicting flu trends (12, 13) in the vein of other research that attempts to use large datasets to predict societal trends.

Unpacking communication practices on Twitter are not easy as the messages are limited to 140 characters and this makes it difficult to get any sense of nuance. For example, sentiment analysis can be misleading as jokes or sarcasm can easily be misinterpreted. In studies exploring the links shared by others, the presence of parody is known to complicate the interpretation of data (11). As a consequence, we argue, it is important to supplement large-scale automated analyses with qualitative approaches that can explore nuance and interpret subtleties that cannot be otherwise detected. To a certain extent, this mixed methods approach was adopted in the Chew and Eysenbach paper, which provides a model for what can be achieved by a big data, little data combination. However, the goals of the study we report here are rather different.

As we noted earlier, compliance with official guidance can be crucial during a pandemic, but the information and advice disseminated by health authorities can be contested and social media has provided a platform for such activity. A major goal of our current study, then, is to use Twitter data to better understand public discourse in the wake of a sequence of key UK-based health announcements about the 2009 H1N1 pandemic. Here, we are focusing upon the responses made by the UK public to an orchestrated series of health press-releases and directives and also exploring the ways in which vaccination and antiviral advice was promoted or contested throughout the network.

In the first part of the paper, we ask simply how the UK data compare to the global data as described by Chew and Eysenbach (3) and also note the variation in Twitter activity around the time of seven key public announcements. In the second part of the paper, we focus more closely upon the impact of these announcements – specifically in terms of the press releases and vaccination guidelines issued by the UK Department of Health – seeking to understand how people interact with this information. In the third part of the paper, we explore in more detail the barriers and facilitators to adhering to the official advice from the UK Department of Health, seeking to fit the data to a theoretical model of human response to threat. This analysis, as a whole, aims to offer a more detailed and nuanced picture of the opportunities and challenges associated with pandemic health-communication on Twitter.

## Materials and Methods

### Data Collection

UK Twitter data were delivered to us from our supplier, Gnip. The data consisted of 14,312 tweets that had been identified by searching the Twitter archive using the following search terms:

((H1N1 OR “swine flu” OR swineflu OR pigflu OR “pig flu” OR “pandemic” OR influenza OR flu) AND (vaccin OR antiviral OR jab OR vacin OR vaccines OR injection OR shot OR Tamiflu)) OR (Tamivir OR Relenza OR Pandemrix OR Celvapan)

These keywords allowed for partial matches, meaning that the word “vaccine” in a tweet would be matched by the search keyword “vaccin.” The tweets were also filtered geographically by using the location information supplied by users in their Twitter profile. The search terms were:

“United Kingdom” OR “Scotland” OR “Wales” OR “Northern Ireland” OR “UK” OR “Great Britain” OR “GB” OR “England”

The Twitter archive was searched for 395 days between 01/04/2009 and 01/05/2010. The geographical search terms resulted in the inclusion of tweets from New England (*N* = 1601), which were subsequently removed from the data giving a total of 12,711 tweets. Most Twitter users supply valid geographical location in their user profile (14), so this should represent the majority of tweets relating to H1N1 during this period. Ethical approval was granted for the study from the Psychology Department at Northumbria University.

### Analytic Approach

We used three different analytical approaches. For the first set of descriptive analyzes, we used multiple tools including R (15) for managing and plotting data, KH Coder (16), a program based on R for content analysis, and Gephi (17), for producing network graphs of Twitter users.

For the second, discourse analysis, we selected a subset of tweets made in the wake of health announcements and press releases from relevant UK authorities, sorting the data by date ranges and keyword criteria. These were analyzed thematically (discursively) with a view to understanding public response to the press releases. Discourse analysis with its focus on the *action-orientation* of language implicitly informed this analysis (18).

In the third, thematic analysis, we used a template analysis (19, 20). This form of thematic analysis stresses the importance of creating an hierarchical arrangement of themes and frequently draws on other theoretical frameworks to provide a deductive (rather than inductive) structure to the themes. In this case, our first analysis of the data suggested that the best fit to the data was a theoretical framework accounting for health-related behavior in the face of a known threat [Protection Motivation Theory (21)].

## Results

### Overall Trends in the UK Twitter Data and Comparison with International Data

#### Trends Over Time

**Figure 1:**
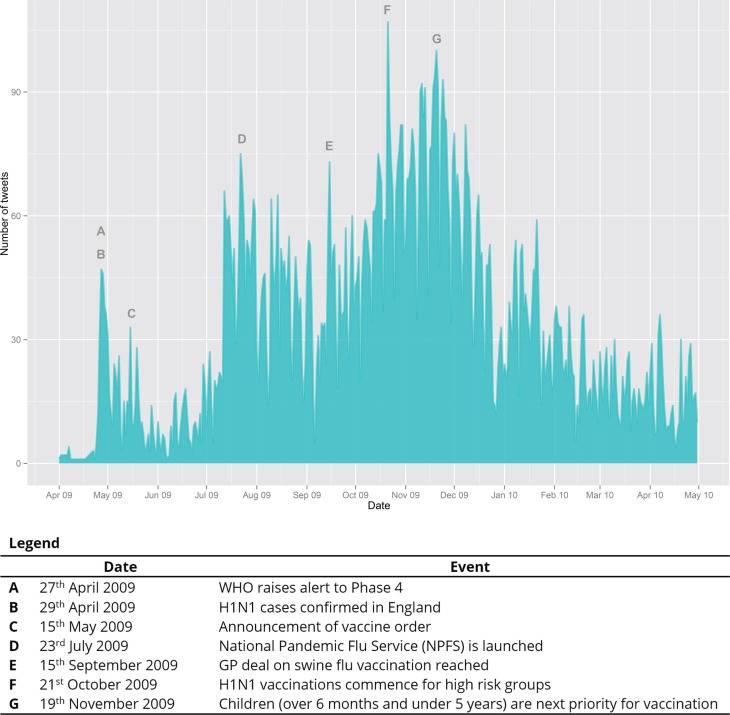
**UK Tweets about H1N1 treatment plotted against time**.

Before exploring the evaluation of health information on Twitter in detail, it is helpful to survey overall trends in the data. Taking the whole dataset and plotting the tweets against time (Figure 1), we can make some general observations. First, comparing this UK dataset with worldwide Twitter data, Chew and Eysenbach (3) reported peaks in late April/early May, mid-June, mid-July, and late-October/early November. These are generally consistent with our data in terms of spikes in activity. Note, however, that the worldwide data showed most activity in May 2009, whereas our data show most activity during October and November of that same year. This difference may be because the vaccine became available from 14th October in the UK, triggering increased discussion of treatment. Second, then, we map the UK Twitter data onto key stages in the progress of the pandemic and its management by health authorities. These are shown in Figure 1 as events A to G and correspond to peaks in Twitter activity, as expected.

Note that the data do not entirely follow the H1N1 case trends reported by Hine (22), where reported cases peaked in early July 2009 and at the end of September/beginning of October 2009. The UK Twitter data show no corresponding peak in September/October. While our keywords restricted us to discussions that explicitly involved vaccination or antivirals, the data suggest that no direct relationship can be posited between cases and discussion about H1N1 treatment on Twitter. Comparison with trends in UK newspaper reporting of H1N1 pandemic (7) reveals a peak in reporting in May that corresponds with Twitter discussions and a peak in July likewise; however, whereas newspaper reporting decreased steadily from October onward, Twitter exchanges remained highly active. Again, with the caveat that our data represent only discussions involving vaccination and antiviral use, there is not a direct correlation between newspaper reporting and discussions on Twitter. This may be because newspapers deal more with upcoming threats and pay less attention to everyday management of the pandemic as is discussed on Twitter.

#### Patterns of Influence in the UK Twitter Data

**Figure 2:**
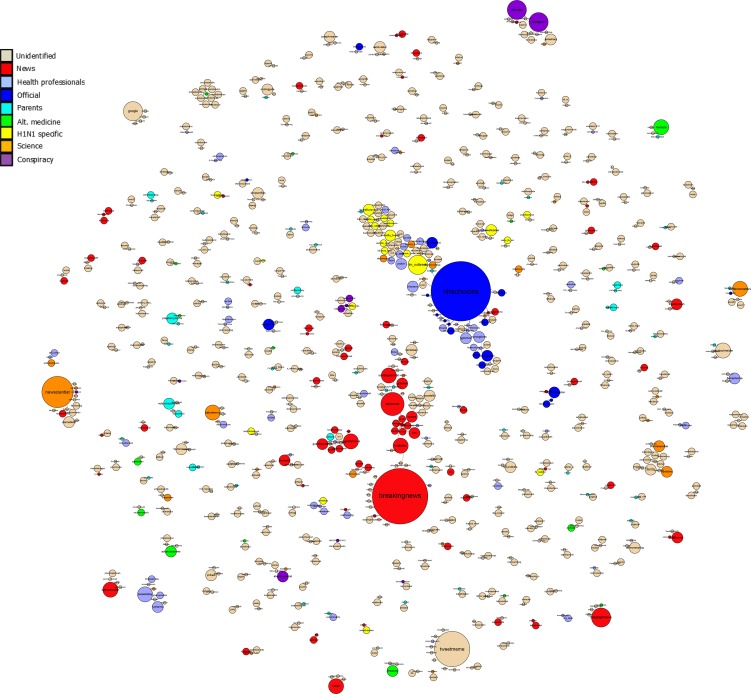
**Network Graph showing user-relationships based on retweet frequency**. Larger nodes represent a higher number of retweets of the source. Users who more frequently retweet the other are closer together.

To further understand the patterns of influence in the overall data, we identified all Twitter retweets and extracted the usernames of both the originator of the tweet and the individual or organization retweeting. Users were also categorized by user type to help interpret the data. We classified users using keywords that indicated their placement in one of the following categories: *News, health professionals, official sources, parents, alternative medicine advocates, H1N1 update accounts, science-related accounts*, and *conspiracy theorists*. These categories were produced inductively by sorting accounts into pandemic-relevant categories. The data were then plotted as a network graph using Gephi (17), shown in Figure 2. The network graph has three main features: the categories above are color coded, the size of circles (nodes) is proportional to the amount of times the user was retweeted and the users who retweet each other more often are closer together.

Figure 2 indicates several features about the sharing (retweeting) of information on Twitter in the UK during the pandemic. Users tend to cluster around two dominant sources: one is the “BreakingNews” account and the other is the “NHSChoices” account. These were the two most retweeted sources in the UK dataset. Note that the “breakingnews” account was influential, but was not a UK-based site. Therefore, the most retweeted account from UK sources was NHSChoices, which suggests that Twitter was an effective vehicle for the dissemination of NHS generated content. Note, however, that the number of tweets provided by NHS Choices was relatively low, which effectively limited their overall influence during this period.

#### Content Analysis of Tweets

To get a sense of the general content of the data, automated content analysis was conducted using KH-Coder, a textual analysis program. The most frequently used words (excluding search terms) and their counts are given in Table 1. The total number of words was 150,723. All words were stemmed before analysis, which means that the words in Table 1 include cognates (e.g., “get” includes “got,” “getting,” “gets,” and so on).

**Table 1:** **Most frequently used words (excluding search terms)**.

Noun		Adjective		Verb	
Health	905	New	626	Be	5525
Shot	832	Seasonal	325	Have	3443
News	803	First	259	Get	1873
Child	516	More	242	Do	1036
Today	477	Free	241	Say	695
Arm	428	Pregnant	221	Take	496
People	395	Good	219	Go	446
Dose	317	Available	210	Give	434
Week	312	Last	176	Make	388

*Numbers indicate frequency*.

With regards to nouns used in the data, the common occurrence of words relating to news (“news” and “today”) suggests that a dominant content of the tweets is news material. Some of the topics related to news emerge in the nouns identified (such as news about children getting vaccinated; e.g., “every child in Scotland to be vaccinated against swine flu”). The presence of the word “arm” in the most frequently used words links to tweets in which people typically talk about how the injection hurt their arm (e.g., “apparently it is not just me who has a sore arm after my flu shot. The whole company walked in complaining about it”).

Common adjectives show that there was frequent reference to the newness of the vaccine and the flu strain. “Pregnant” women getting vaccinated was a topic that also occurred regularly since they were an at-risk group who were advised to take the vaccine (e.g., “pregnant women front of line for swine flu vaccine”). Words such as “available” relate to the availability of treatment and statements about when and how people can access treatment. The word “sore” regularly refers to vaccine side effects such as pain in the arm. Verbs indicate the level of references to people talking about doing things in relation to H1N1 treatment. Thus “get” and “take” indicates that a large number of the tweets refer to people getting and taking treatment.

While this is only a general overview, these keywords give some insight into the most regularly occurring content on Twitter during the pandemic and most of this seems to be news related. Twitter is widely recognized as a site for sharing news, and this is confirmed in this overall content analysis. However, the use of words like “get” and “take” indicate that there are some tweets that are not news-related but deal with the personal experience of treatment.

#### Websites Referenced

In the last part of this general overview of the content, we examine the websites most frequently referred to in the tweets. Because Twitter is widely used for disseminating news or information and because of the forced brevity of the messages, many tweets link to other articles. In our dataset, we found that 8414 out of 12,711 tweets contained URLs (66.19%). We extracted these URLs from the tweets, un-shortened them, and parsed them to find the host name. The most frequently referenced hosts are shown in Table 2.

**Table 2:** **Most frequently referenced websites**.

Host	Count	Type of site
www.bioportfolio.co.uk	497	Biotechnology news
bit.ly	336	URL shortener
news.bbc.co.uk	302	News
www.youtube.com	271	Video sharing
www.swineflunews.org	248	H1N1 news
cli.gs	186	URL shortener
tinyurl.com	130	URL shortener
www.google.com	125	Search engine
www.swine-flu-news.com	123	H1N1 news
www.NaturalNews.com	114	Antivaccine
www.theguardian.com	113	News
www.telegraph.co.uk	112	News
drop.io	92	File sharing
www.reuters.com	79	News
www.officialwire.com	78	News
www.dailymail.co.uk	78	News
swineflunewswire.com	75	H1N1 news
www.barbicanacupuncture.com	74	Alternative medicine
www.earthtimes.org	64	Green news
www.examiner.com	56	News

Most of the sites represented in the list of top URLs are general news sites (*N* = 938 tweets) and most of these contained reliable information about the pandemic (i.e., consistent with health authority advice). When all news sites are considered, there are 2042 news sites referenced. There are some antivaccination sites mentioned (*N* = 218; conspiracy and antivaccine), which represents a small but vocal minority.

**Figure 3:**
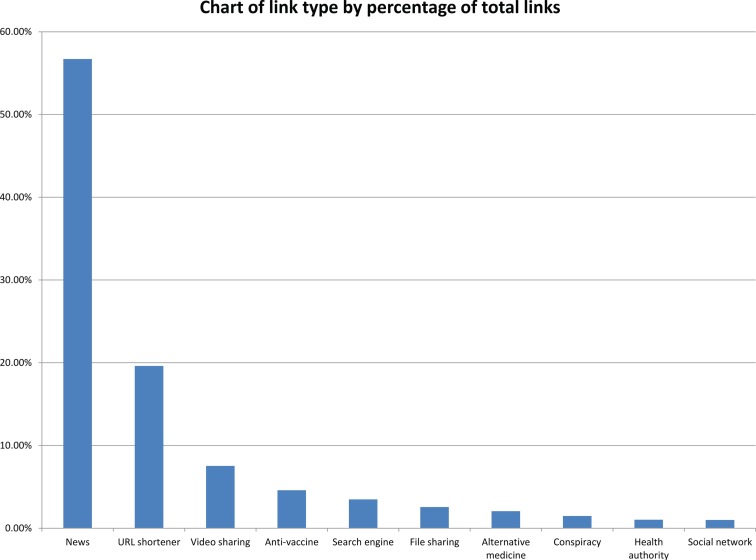
**Chart of link type showing percentage of total links**.

The percentage of each type of link is represented in Figure 3. Comparing this with the analysis of links from Chew and Eysenbach (3) suggests that there are similarities between the UK data and the global data in the percentages of news websites, health authority sites, and social network sites. However, they did not code for antivaccine sites, alternative medicine sites, or conspiracy sites. Including these in our analysis of links shows that while there was a predominance of information generally positive about the recommended treatment, there was a vocal minority that opposed the vaccination.

Overall, the high proportion of tweets containing links reinforces the concept of Twitter as a news-sharing network. However, there is a significant quantity of tweets without any links, and these are more likely to contain users’ evaluative comments rather than simply links and descriptive headlines.

### Public Responses to Press Releases

Having surveyed overall trends in the data, we observed that a high proportion of tweets seem to be linked to news-related information. We were particularly interested in understanding how people respond to such information. To do this, we selected three events from the timeline that were accompanied by press releases from the Department of Health: (1) The order of the vaccine (Figure 1, C), (2) The deal with GPs on administering the vaccine (Figure 1, E), and (3) The announcement of the commencement of the vaccination program (Figure 1, F). By selecting events accompanied by press releases, we were able to identify the key terms used in the event and then to search for tweets talking about those events.

#### The Vaccine Order (15th May 2009)

On 15th May, the UK Department of Health released a statement saying that, “Agreements have been signed between the UK Government and vaccine manufacturers to secure supplies of up to 90 million doses of pre-pandemic H1N1 vaccine before a pandemic begins” (23). To explore what people said about this, we search tweets from 15/05/2009 to 31/05/2009 using the keywords “order,” “90m,” “buying,” “agreement,” and “secured.” In total, 38 tweets were selected matching these criteria from a total of 181 in that time period. This number is quite small and can be accounted for by noting that Twitter was still a relatively new social networking site (<3 years old) and that our initial search criteria were quite specific. Taking these 38 tweets, we excluded 5 because they were about vaccine orders for other nations leaving 33 tweets. Of these, 23 were neutral (descriptive, news-reporting), 2 were positive, and 8 were negative. An example of a typical descriptive tweet is, “*Deal on 90m UK swine flu vaccines [website link]*.” Because we were specifically interested in evaluation of the information, we examined the 10 evaluative tweets to see how they responded to the information.

A common feature of the negative tweets is that they make attributions regarding the government’s announcement of the vaccine order either by direct accusation or by implication. Implied attributions are those that question some aspect of the information so as to get the reader to attribute thoughts or abilities to the referent. These attributions often take the form of questions in which the user questions the *rationale* for ordering so many vaccines (“*If there are 65m people in the UK, why has the govt ordered 90m swine flu vaccines?*”) or the *adequacy* of the preparation (“*Alan Johnson: “Enough #swineflu #vaccine to protect 1/2 of the #uk population by December*.” *What if #pandemic comes in September?*”). These questions encourage the reader to form their own personal attribution of the rationale or adequacy of the government’s preparation by using rhetorical questions. At other times, simple statements are employed to allege that the government has improper motives for ordering the vaccine: “*scare mongering works! Scotland buying enough swine flu vaccine for the entire population*.” Of the eight negative tweets, seven expressed explicit or implied attribution toward the government (or media reporting the announcement in one case). Of the two positive tweets, one stated personal expectation of receiving a vaccine while the other stated that the government was well-prepared for the winter flu (another attribution).

While this is only a small number of tweets, the dominant feature was the way users attributed cognitions and preparedness to the government. There has been extensive research into the process of attribution (24–26). Potter et al. (27) avoid cognitivist explanations of attribution and focus on the rhetorical function of attribution-talk in conversation. From this perspective, attributions are considered in their social and rhetorical contexts in order to understand why they are being used and to what ends. In this case, attributions of the government’s rationale, for example, appear to be made for the purpose of deriding, or at least calling into question, the decision to order 90 million vaccines. Although we cannot be certain about the extent of influence this has among other users, source credibility judgments are affected by attributions of the source [specifically, attributions of motive (28)]. Consequently, trust in advice from the government will be linked to the kinds of attributions people make about the source.

#### The GP Deal (15th September 2009)

The next incident of interest is the announcement by the then Health Secretary, Andy Burnham, of a deal for GPs to administer the vaccine to their patients for a payment of £5.25 per vaccine. This was reached after negotiations between the Department of Health and the General Practitioners Committee. To collect tweets on this issue, we searched tweets between 14/09/2009 and 30/09/2009 for the keywords “gp,” “deal,” and “5.25.” After removing some irrelevant results, this produced 23 tweets. However, once simply descriptive tweets were removed (tweets simply reporting that the deal had been struck), only five tweets were left that included some form of evaluation of the announcement.

Three of these tweets questioned the GPs’ motivation and implied that they were motivated by financial concerns. One of these tweets was apparently sarcastic: *“#swineflu - that’s disgusting - £5.25 for every jab doctors give - not even making minimum wage!”* Such tweets derogate the motives of the doctors by drawing attention to the money they are being paid to vaccinate. Another tweet questioned the fairness of government spending by comparing it to budget cuts elsewhere: “*Health boards face Scottish government’s £500m budget cuts yet GP’s to be paid £5.25 for every dose of flu vaccine they give to patients*.” One other user (a medical account) questioned the fairness of the deal implying that doctors were not getting paid enough: “*GPs to be paid £100m for giving swine flu vaccine. Is £5.25 for every dose a fair deal?*”

All of these tweets involved an attribution. Three of them attribute motives to the doctors and two attribute unfairness to the government. As in the previous case, these attributions either supply unstated information or change supplied information in the announcement. In the case of motives, the motive of financial gain is supplied while the characteristic of fairness is manipulated, since the original announcement talked about the “value for money” of the deal and the “fair deal” that had been reached. While the issue of financial motivation in this announcement may not be picked up extensively, other tweets mention financial motives and, as previous research indicates (29), attributions of financial motivation can be a major disincentive for vaccination.

#### The Commencement of the Vaccination Programme (21st October 2009)

In October 2009, the Department of Health announced that the vaccination program had commenced. This announcement delineated the first people to receive the vaccine: frontline healthcare workers, people at risk of seasonal flu, all pregnant women, and household members of immunocompromised people. We searched tweets from 20/10/2009 to 15/11/2009 using the search terms, “risk,” “priority,” “groups” and “special.” The search returned 65 tweets of which 47 were descriptive (e.g., *“swine flu vaccination programme begins in British hospitals today for staff and high risk patients - official. #swineflu #h1n1 #flu”*) and 14 were evaluative.

In this set of 14 tweets, 13 users were eligible for the vaccine and 1 was not (“*we have the swine flu vaccine in the UK but we stand no chance of getting it, we are bottom of the priority list!”*). Of the 13, only two personally affirmed that they were high risk. The other 11, through various means, questioned being “high risk” as labeled in the announcement or letters from their GP. One regular way of questioning this identity is through the use of quotation marks: “*Having a flu jab this lunch time - apparently I’m an ‘at risk’ person because I got given an inhaler over the summer. Ludicrous*.” These quotation marks suggest the user has not embraced the identity of being “at risk” but sees it as an external imposition. Another user explicitly notes the external nature of this identity and expresses an element of dislike for it: “*Got an invite from my GP to have a swine flu jab. Am not sure I like being categorised as in a ‘priority group’. Makes me feel decrepit*.” Still another way of emphasizing the externality of the identity is by showing lack of knowledge regarding the meaning of the identity: “*I have to get the swine flu jab tomorrow because I am at risk! Or something*.” The words “or something” express a lack of awareness over the imposed identity and a lack of personal ownership of the label.

These tweets show that a large proportion of those evaluating the information saw the identity of being “at risk” as being something externally imposed rather than something that they personally understood and accepted. In a way, they were attributing their identity to the viewpoint of the health authorities or medical professionals. In contrast to the previous two press releases, where users attributed features to the health authorities, here the users attribute cognitions *about themselves* to the health authorities. This externally imposed identity then, is not fully accepted by some of the users and while this does not necessarily lead to mistrust or refusal to get vaccinated, it does seem to be linked to uncertainty. For example, one user says, “*Just got my swine flu letter! I’m a priority! Is that good or bad? To jab or not to jab, that is the question. Whether to suffer misfortune!”* The uncertainty about accepting the identity and its meaning is linked to uncertainty about whether the vaccine should be accepted or not.

#### Summary of Public Responses to Press Releases

While the majority of tweets to refer to the content of the press releases were purely descriptive, some tweets were evaluative. Such evaluation is interesting in light of the potential consequences of “recontextualization” (30) in which information is reproduced in a different context, which changes the meaning and effects of the original message. In this case, the addition of attributions to the original intent of the messages may have the effect of altering trust in the referents of the information. Attributing unpreparedness to the government because of the timing of vaccine deliveries or attributing ill-motives to doctors are examples of how attributions may affect trust. Furthermore, attributing an attitude toward health authorities that perceives them as arbitrarily labeling people as “at risk” may have implications for readiness to receive a vaccine. Analyzing how information is disseminated, as we have done here, facilitates understanding how messages can be received or even distorted by the general public.

### A Theoretical Framework to Capture Public Health Discourse

Where tweets related to press releases, the majority of tweets functioned to spread informational messages from other news sites. This is consistent with other research that suggests Twitter is largely a news-sharing network (31, 32) and with the finding that around 53% of tweets about H1N1 globally were “resource” type tweets (3). However, our sample did contain a number of twitter “conversations” in which tweets had a more evaluative context and which were more illustrative of the ways in which different individuals might interpret or even challenge the information and advice that was circulating on Twitter.

A Twitter conversation is indicated by placing the “@” symbol before a username and this functions as a form of address. These conversations are easily identified and are generally conversational, rather than descriptive, in content. For our analysis, we collected 1164 tweets that could be identified as conversations and then identified a further 412 tweets that would inform our understanding of the barriers and/or facilitators for vaccination and antiviral use. We used these conversations as a means of understanding more about uptake and non-uptake of vaccines and antivirals during the pandemic. In other words, we explored the ways in which public health information may be both shared and contested on Twitter.

For this analysis, we used a template analysis (19, 20) that allowed us to explore the fit between the data and known theoretical models of health behavior [see Ref. (33) for a review]. We used Protection Motivation Theory [PMT; (21)] as it has been shown to be an effective model in predicting responses to pandemic flu (34) and because it has two important components that seemed a good match to our initial analysis of the data: (1) a threat appraisal made in terms of both severity and vulnerability to threat and (2) a coping appraisal that contains both an assessment of the efficacy of the coping mechanism (vaccine) and also the cost of making a response. Thus PMT provided a coding framework that was able to capture four important elements in the data: first, the public’s appraisal of the severity of the threat; second, their beliefs about their own individual susceptibility (and that of their friends and family) to the H1N1 virus; third, their beliefs about the efficacy of the measures put in place by the health authorities (in this case, beliefs about the efficacy and availability of the vaccine and antiviral medication) and fourth, their perceptions around the “cost” of receiving appropriate medication in terms of both the “cost” of going to the doctor (e.g., taking time off work) and also the health and wellbeing “costs” of vaccination (including fears of an adverse reaction to the vaccine and fear of needles). These are described in more detail below.

#### Judging the Risks Associated with the Pandemic (Threat Appraisal)

Threat appraisals are central to several theories of health behavior (35) and have been shown to be a critical element of judgments made about vaccination (36). In Protection Motivation Theory (21), these appraisals involve judgments about the overall severity or seriousness of a hazard (the H1N1 pandemic in our case) and individual susceptibility or vulnerability to that hazard which, in combination, provide a potential trigger for action. We discuss these judgments separately.

##### Perceived Severity

In a meta-analysis of factors that predict the uptake of flu vaccination, Brewer et al. (36) showed perceived severity of the flu epidemic had a low-to-moderate, but nonetheless significant, predictive effect. They noted, however, that the relationship appeared stronger in studies assessing the views of clinicians as compared to studies assessing the views of the general population. In our qualitative analysis, we found a similar disparity. Relatively few personal tweets displayed anxiety about the overall severity of the pandemic, but several stated that their nurse or doctor had urged them to take action in the face of the pandemic in part based on the professional’s severity perceptions:

I want the vaccine, despite the bad press it’s been getting. My doctor said swine flu is very serious :(

my daughter recently had swine flu total angst re tamiflu. i had good nhs direct advice and gave it to her. over in 4 days

top bod on pandemic flu committee just recommended i go out and get me some tamiflu (despite unattractive name)

However, more typical of our sample, were conversations that suggested that “too much fuss” was being made of the outbreak. Such conversations were often linked to a refusal to consider taking the vaccine or antiviral medication:

*wouldn’t stress about swine flu,90% of my friends had it, its not much worse than a normal flu.dont take tamiflu,take vitc&d*+*garlic*

swine flu is not a severe illness for most ppl

*given that this is not half as bad as flu i’ve had before, i’m inclined not to bother with antiviral anyway*...

*largely pointless, tiny risk of complication, less dangerous than most flu*.

*oh please don’t. i am getting so fed up of the hype surrounding swine flu*.

Note that one of the emergent factors, which seemed to underpin the difference between clinician assessments of severity and those of the general public, concerned trust. For a number of the tweeters, the information and advice about the severity of the pandemic was compromised – sometimes by a perceived desire for government to “sell off the vaccine” (and related to press releases about the vaccine order and GP deal, noted above).

*It’s like normal flu, but dramatic and frightening. I bet the people behind Tamiflu are making billions off the scare tactics*.

hmm it’s so not swine flu vaccine, is probably money in the needle...injections of cash :D

*swine flu is part of a conspiracy to sell tamiflu. there was a surpluss after bird flu didn’t take off*.

Overall, we gained a sense that H1N1 influenza was perceived as a fairly mild disease and that too much fuss was being made about it. This is consistent with the findings of a systematic analysis of the literature on the uptake of vaccination for pandemic influenza (34), which indicated that citizens in the UK (37), the USA (38), Canada (39), and Australia (40), were likely to regard the severity of the pandemic as similarly overblown.

##### Perceived Vulnerability

In both the meta-analysis (36) and the systematic analysis (34), vulnerability perceptions were more reliable predictors of vaccination uptake than were severity perceptions. Again, we see this reflected in our own data, where discussion concerned pre-existing conditions that were likely to affect individual susceptibility:

h1n1 and pregnancy can be more problematic

I automatically qualify for swine flu jab as I’m in a “high risk category” (asthmatic)

i spent 6 months with my lungs knackered after a bout of ‘flu five years ago. i hassle my gp for the jab every single year now :)

While vulnerable individuals promoted the idea of vaccination, there was a strong sense that it might not be necessary for otherwise healthy individuals:

@sarknight #swineflu jabs - what do you reckon? survey among nhs nurses showed 50% would not have the jab. not needed if in good health

Here, we see a sense that, consistent with PMT, individual vulnerability was recognized as being critical. When severity perceptions were also high, this triggered readiness to seek vaccination.

#### Assessing Ability to Mitigate Risk via Vaccination or Antivirals (Coping Appraisals)

An important element of PMT concerns the ways in which people make judgments about whether or not they can actually take the necessary remedial action. In other words, people make a coping appraisal which, in turn, involves a number of discrete judgments: about whether or not the medication would prove effective (response efficacy); whether or not they would have access to medication (response availability), and also about the *costs* of medication, such as the side effects of the vaccine and antivirals, the affective costs (e.g., fear of needles), and the time costs (inconvenience).

##### Perceived Efficacy of the Medication

Investigations have shown that vaccination uptake is predicated on perceptions of vaccine effectiveness, i.e., that it will reduce the individual’s chances of catching H1N1 influenza (34, 41). We observed doubts about both the efficacy of the vaccine and also significant confusion between the vaccine and the antiviral, Tamiflu:

*@danwood @bbum @danielpunkass pharmacist friend says that (over here) there’s been much less trialling than normal for h1n1 vaccine*.

you even want your kid to get the swine flu shot? it’s not been tested:/

if it is about h1n1, the jab is new and people are understandably a bit suspicious, and swine flu is not a severe illness for most ppl

*tamiflu does help, will shave a couple of days off if its swine flu*.

If your dad has swine flu I’m coming round and injecting you with Tamiflu

The muted antiviral response was exacerbated by some published reports that a Tamiflu-resistant strain of H1N1 had emerged and others that suggested that Tamiflu only reduced symptoms by around 1 day (42). This led some to conclude that it would be ineffective at treating or preventing H1N1. In fact, subsequent claims were mixed. In one study, hospitalized adults were found to be 25% less likely to die as a result of the drug (43), but a 2014 Cochrane review that used data from 20 trials of oseltamivir (Tamiflu) concluded that its impact was indeed modest – with some alleviation of symptoms, but no impact on either the prevention of pneumonia or in terms of disrupting the spread of the disease (42).

##### Perceived Availability of the Medication

There was a significant discussion about the availability of both the vaccine and the antiviral medication, with some debate about whether the drugs would be reserved for only the most vulnerable:

we have the swine flu vaccine in the uk but we stand no chance of getting it, we are bottom of the priority list!

*least your getting the swine flu jab, i*’*m gonna be last on the list, i bet you!*

re:tamiflu you can do it online, google swine flu nhs should find it easily, they give you a code you have to use

*oh no, hope you haven’t got #swineflu. only certain chemists in leicester stock tamiflu*,

In fact, UK Government advice was that all high-risks group should receive a vaccine, but there was some additional confusion as two vaccines were made available which differed in terms of the presence of an adjuvant to stimulate an immune response. This acted as a further barrier to action as people were unsure about the most appropriate choice:

Actually, I believe that the swine flu is just one jab unless child has egg allergy, then they need a different vaccine

Oh you had the flu jab? Is it okay so far? My family are having them next week, I cant, I’m allergic to eggs!

In this last case, the belief about allergy-related ineligibility is only partially correct. Certainly the non-adjuvanted vaccine Celvapan (made by Baxter) was widely available for people with egg-allergies; perhaps better communication concerning this may have overcome such confusion.

##### The “Response Costs” of Medication

Consistent with PMT, by far the most significant issue in our Twitter dataset involved the weighing up of the costs and benefits associated with the taking medication. The dominant issue was the safety of the vaccine. Tweets were evenly split between those that said it was safe and those that said it was unsafe, but the commoner concerns were with the short-term side effects which, for some people, were quite severe and appeared to create a significant barrier to uptake.

1976 #swine #flu: 300 infected 1 died. Of 40m vaccinated: 25 died, 500 paralysed. Jab/shot program abandoned. Comments?

I took Tamiflu last week cause I had swine flu....sick as dog...and hallucinating seeing spiders and stuff

Have you got the swine flu? It’s a killer. Tamiflu makes you feel sooo much worse. You have been warned :(x

stay clear of tamiflu - it makes you vomit

@kelliente i got the h1n1 shot b/c i have asthma...not a single side effect...though i now will likely become zombie on dec 20, 2012...damn

Such barriers to uptake being spread throughout Twitter may have contributed to a broader concern about Tamiflu, which seems to be reflected in the levels of unused antivirals in the UK (44). Certainly, the ready availability of peer generated information via Twitter seemed to increase the likelihood that health decisions would show a strong peer influence, reflecting an established eHealth finding that people would often turn directly to others for information and advice (45):

u havent had ur swine flu jab have u? i was just wondering cos I wasnt sure whether there wer bad symtoms after?

*u get any replies about the swine flu jab cos my little man needs his*.

The *anticipated* emotional cost of medication was often high, tied to a fear of needles or of unknown reactions to the medication. This fear response was seen more often in those who were worried about the consequences of vaccinating a child. In several circumstances, fear of the drug or its administration overrode the fear of getting the virus itself:

*i need a flu shot.... but i*’*m terrified of needles!*

*no, i*’*m not having a swine flu injection, scared of needles and sceptical about injections/side effects*

not good! sophie was supposed to go get the swine flu jab and i wont take her, im to worried about it

I am more scared of taking Tamiflu than the flu itself. Why do they recommend I take it I wonder?

Finally, we also saw some discussion of ways to offset the physical costs or inconvenience of acquiring medication, both in terms of using the National Pandemic Flu Service helpline to access antiviral medication (46) and also in the recruitment of “flu friends” who could help obtain the medication. Both of these initiatives had the impact of keeping sick people at home and thereby limiting the penetration of the virus, although it is worth noting here that while various forms of isolation can help to contain an influenza outbreak, in the longer term, such isolation can also be viewed as a response cost (47).

Only flu friends should pick up Tamiflu

*well, we*’*ve got 1 flu friend each then, pip will have to run between houses with a little barrel of scotch and tamiflu*..

my aunt and uncle also got #swineflu diagnosis over the phone, had to get mate to pick up tamiflu prescription

Overall, then, in this final conversational analysis, we have seen how Twitter can be used to communicate both a social and an emotionally evaluative response to the pandemic. This contrasts sharply with the more neutral descriptive and informative content coming from healthcare providers and helps us understand a little more about how the perception of threat and the ability to deal with threat enter into the health equation.

#### Summary of a Theoretical Framework to Capture Public Health Discourse

In using template analysis, we were able to match the data to Protection Motivation Theory which helped to explain how people managed the threat of the pandemic. Appraisals of the threat focused less on overall severity and more on personal vulnerability. However, such threat was managed by coping appraisals of the efficacy of the treatment, availability of treatment and the response costs associated with receiving a vaccine or antiviral. While such themes are not intrinsically novel, exploring the themes reveals more specific features such as the influence of short-term treatment-effects on decisions to accept treatment. Using a detailed qualitative approach shows specific aspects of decision-making that might normally be lost in aggregate data.

## Discussion

The aim of this analysis was to produce a more detailed picture of how Twitter is used to communicate health information. In the first stage of the analysis, we explored the data overall. We suggested that the trends of the data tend to correspond, in some respects, to public health press releases (which in themselves marked key events). We observed that the main UK source to be retweeted was NHS Choices, which signaled that even in this new social media environment people were turning to the health authority for information. Most of the other retweeted sources were reputable news organizations. The overall analysis suggested that most tweets were descriptive and had the function of sharing news information, although there were also a large number of personal experience tweets. The URLs embedded in tweets were mostly links to news websites, while some antivaccination sites were present.

In the second part of the analysis, we explored the communication of press releases on Twitter. We found that most of these tweets were descriptive, although there were a small number of evaluative tweets. These tweets tended to make attributions about either people or agencies mentioned in the original press release and these gave us some useful insights into the critical reception of press releases disseminated by the public on Twitter.

In the third part of the analysis, we explored in detail conversations about vaccines and antivirals to understand the barriers and facilitators of vaccine/antiviral use. Using Protection Motivation Theory (PMT) as a framework, we saw the way that peer dissemination of information in relation to both the overall threat of the pandemic and the cost of taking the vaccine/antiviral played an important role in affecting decisions about uptake of the vaccines/antivirals.

Numerous points can be drawn from this. First, Twitter was largely used for sharing news information (31). This may serve the function of creating awareness of the pandemic and raising general levels of risk from the virus and its treatment. Just as media is often accorded an *agenda-setting* function by promoting certain topics (48), Twitter may promote certain stories as being of particular interest and relevance to the general public.

Second, the information tweeted and retweeted between members of the public often cited reliable sources of health information such as NHS Choices or the more “trusted” news websites such as the BBC. There was no sense that the democratization of influence on Twitter during the pandemic led to the circulation of health messages that were radically different from those promoted by the UK health authorities. Even those concerns that circulated about the effectiveness of the available medication were consistent with subsequent large-scale analyses of Tamiflu and other antivirals (42). We saw very little spreading influence of antivaccination lobbyists – a finding that reflected in our third analysis, where barriers to vaccine-uptake were expressed in terms of short-term risks such as sickness and pain rather than serious long-term risks such as autism or Guillain–Barré syndrome.

Third, in our analysis of Twitter responses to key press releases, we have seen that people like to reason about *why* they are given certain information. This reasoning can, in turn, underpin judgments about whether to trust vaccination information and advice. While we saw a lot of respect for the views of health practitioners, consistent with that observed in other studies (49), the suspicions generated by an economic argument (that GPs were being paid to vaccinate or that profits were being made from selling off a Tamiflu surplus) sometimes undermined the official position. We already know that, to be successful, health interventions should originate from a trusted source such as a GP or public health body (50), but here we also see how easy it is to publically undermine the motivations of those in that trusted position.

Finally, the use of the PMT framework highlighted some of the ways in which health communication could be made more effective. For example, while the “too much fuss being made” argument (49) was ultimately seen as realistic for this pandemic, future social media analyses could provide early alerts to this type of public response. Perceptions of personal vulnerability could have been made clearer and public confusion about the role of vaccines and antivirals led to doubts about the response efficacy of taking either.

### Limitations

The predominantly qualitative nature of our approach is both a strength and a weakness. While it allows manual coding of tweets to accurately reflect their content and while it allows detailed analysis of what is said, inevitably it cannot provide quantitative evidence for the extent of antivaccination sentiment or vaccine uptake (for example). Indeed, the applicability of quantitative methods to some of our analysis (see Public Responses to Press Releases) is minimal due to the low numbers involved. Such low numbers reflect the early nature of Twitter (only 3 years old at the time of the pandemic), the sampling of the data (UK vaccine/antiviral-related data only), and the specific messages being studied (regarding press releases). While these low numbers limit quantitative analysis, they allow detailed qualitative analysis to explore *how* people are expressing themselves both in terms of content and rhetorical strategy.

## Conclusion

Overall, this study shows that there is benefit in analyzing Twitter data, both in real-time as a source of current beliefs and attitudes and as historical data to understand the beliefs that may have influenced vaccine and antiviral uptake. Twitter functions as a news site for creating awareness – but in addition, it provides an arena in which users can share their concerns about health. This provides an ideal opportunity for researchers to investigate these concerns and to explore the use of social media influence in promoting successful behavior change interventions.

## Author Contributions

PB was the local principal investigator and lead academic for this part of the grant award. She led the design of the study, providing expertise in social media and eHealth and made a significant contribution to both the data analysis and the writing of the final paper. AM is the post-doctoral researcher who led the collection and analysis of data and wrote the first draft of the paper. PH contributed to both the design and analysis of the paper and also the theoretical underpinnings when making a decision about the analysis framework. He also co-edited the final paper.

## Conflict of Interest Statement

The authors declare that the research was conducted in the absence of any commercial or financial relationships that could be construed as a potential conflict of interest.
